# Chemokine and Cytokine Receptor Signatures Associated with Pulmonary Endothelial-Conditioned Melanoma Invasion

**DOI:** 10.3390/biom16101510

**Published:** 2026-10-17

**Authors:** Viktória Koroknai, István Szász, Marwa Hamdan, Ágnes Berecz, Margit Balázs

**Affiliations:** 1 Department of Public Health and Epidemiology, Faculty of Medicine, University of Debrecen, 4028 Debrecen, Hungary; koroknai.viktoria@med.unideb.hu (V.K.); hamdan.marwa@med.unideb.hu (M.H.); 2 HUN-REN-UD Public Health Research Group, Department of Public Health and Epidemiology, Faculty of Medicine, University of Debrecen, 4032 Debrecen, Hungary; szasz.istvan@med.unideb.hu (I.S.); berecz.agnes@med.unideb.hu (Á.B.); 3 Doctoral School of Health Sciences, University of Debrecen, 4032 Debrecen, Hungary

**Keywords:** pulmonary endothelial cells, melanoma, invasion, chemokines, receptors

## Abstract

**Background:** Pulmonary endothelial cells are increasingly recognized as active regulators of organ-specific metastasis; however, the mechanisms by which they influence melanoma invasion remain incompletely understood. **Methods:** Six primary melanoma-derived cell lines were first assessed for their invasive responses to HPMEC-conditioned medium. In a separate HPMEC co-culture invasion model, invasive and non-invasive melanoma subpopulations were isolated and analyzed for the expression of 88 chemokine and cytokine receptor genes. Candidate receptors were subsequently validated in primary melanoma tissues (*n* = 15), while their corresponding ligands were analyzed in patient plasma samples (*n* = 30). **Results:** HPMEC-conditioned medium enhanced invasion in half of the melanoma cell lines. Exploratory receptor-expression analyses identified candidate genes, including *CCR2*, *IL11RA*, *IL15RA*, *TNFRSF11B*, and *TNFRSF12A*, with expression patterns potentially associated with the observed invasive responses, while *CCR4* expression was consistently elevated in all invasive subpopulations. In an exploratory analysis of a small primary melanoma tissue cohort, *CCR2* and *TNFRSF11B* expression was significantly higher in tumors from patients with lung metastases, while *IL11RA* expression was upregulated compared with that in non-metastatic tumors. Melanoma cells consistently induced endothelial IL-8 expression, whereas plasma concentrations of the corresponding ligands did not differ significantly between patient groups. **Conclusions:** The elevated expression of *CCR2*, *IL11RA*, and *TNFRSF11B* observed in primary melanomas associated with lung metastases suggests that these factors may contribute to the development or progression of pulmonary metastases. These findings highlight the potential role of bidirectional communication between endothelial cells and melanoma cells in metastatic progression.

## 1. Introduction

Distant metastasis, defined as the dissemination of primary cancer cells to distant organs, is the leading cause of cancer-related mortality [1]. The metastatic cascade involves a series of sequential steps, including local invasion, intravasation, survival in the circulation, extravasation, and colonization [1,2]. Recent advances provide insight into these metastatic steps, including the crosstalk between the disseminated cancer cells and their host environment. The concept of the pre-metastatic niche (PMN) refers to a permissive microenvironment established by the primary tumor before the arrival of metastatic cells at the metastatic site [3,4]. Through the secretion of cytokines, growth factors, extracellular vesicles, and other soluble mediators, primary tumors can remodel distant tissue microenvironments and promote organ-specific metastasis. Preventing the establishment of the PMN has been shown to reduce cancer mortality and enhance the efficacy of immunotherapy in preclinical models [5,6].

The lung is a common metastatic site for a wide range of malignancies, including breast, colorectal, and prostate cancers, as well as melanoma [7]. Tumor-secreted cytokines and chemokines, including transforming growth factor-β (TGF-β) and tumor necrosis factor (TNF), were among the first mediators identified as key regulators of pre-metastatic niche formation in the lung [8]. Reciprocal communication between tumor cells and stromal cells through cytokine–receptor interactions contributes to endothelial activation, extracellular matrix remodeling, and the establishment of a permissive microenvironment for metastasis [7,9]. A well-established example is the chemokine C-C motif ligand 2 (CCL2), which increases pulmonary permeability, promotes the recruitment of bone marrow-derived cells (BMDCs), and facilitates clot formation, thereby enhancing the extravasation and subsequent colonization of circulating tumor cells (CTCs) in the lung [10,11,12].

Conversely, circulating tumor cells are actively recruited to the newly established pre-metastatic niche by chemokines secreted by endothelial and stromal cells, which can guide their migration to specific target organs. Lung-derived chemokines, including CXCL12 (also known as stromal cell-derived factor-1, SDF-1) and S100A8/A9, promote tumor cell migration through receptor–ligand interactions [13,14,15,16]. CXCR4/CXCL12 axis is one of the best-known mechanisms driving the homing of the CXCR4-overexpressing tumor cells to CXCL12-enriched niches [17].

Despite substantial advances in understanding chemokine-mediated metastasis, the mechanisms underlying pre-metastatic niche formation and recruitment of metastatic cells remain incompletely understood. Therefore, elucidating the roles of cytokines, chemokines, and their receptors in pulmonary metastasis may reveal novel therapeutic targets to prevent or interfere with metastatic progression.

In the present study, we investigated whether soluble factors secreted by human pulmonary microvascular endothelial cells (HPMECs) influence the invasive behavior of primary tumor-derived melanoma cells. Furthermore, we examined whether exposure to the pulmonary endothelial microenvironment induces distinct invasion-associated receptor expression signatures that may contribute to pulmonary metastatic colonization. In contrast to our previous studies examining receptor-expression patterns associated with liver metastases, the present study focuses on the response of melanoma cells to the pulmonary endothelial microenvironment and examines whether the HPMEC-associated invasive phenotype is associated with candidate receptor expression pattern and metastatic outcomes.

## 2. Materials and Methods

### 2.1. Cell Lines and Cell Culture

The human malignant melanoma cell lines (WM793B, WM1361, WM278, WM902B, WM1366, and WM3248) were obtained from Coriell Institute for Medical Research (Camden, NJ, USA) and were maintained in RPMI 1640 medium (Lonza Group Ltd., Basel, Switzerland) supplemented with 10% fetal bovine serum (Gibco, Carlsbad, CA, USA) in a humidified incubator (5% CO_2_) at 37 °C. Table 1 summarizes the origin and the mutation status of melanoma cell lines.

Human pulmonary microvascular endothelial cells (HPMECs) were obtained from ScienCell Research Laboratories, Inc. (Carlsbad, CA, USA) and cultured according to the manufacturer’s recommended protocol. HPMECs were maintained in Endothelial Cell Medium in culture vessels coated with fibronectin (2 μg/cm^2^) at 37 °C in a humidified atmosphere containing 5% CO_2_. Once the endothelial cells reached 90% confluence, the culture medium was replaced with fresh medium, and cells were incubated for 48 h. The conditioned medium was then centrifuged at 2000× *g* for 15 min and filtered through a 0.2-µm filter.

### 2.2. Melanoma Tissue and Plasma Samples

Melanoma tissue samples were collected from patients at the Department of Dermatology, Faculty of Medicine, University of Debrecen (Debrecen, Hungary) who had not received any prior treatment for the primary lesion before surgical excision. For each patient, relevant clinical parameters were recorded, including age, sex, histological subtype, Breslow thickness, ulceration, anatomical location, Clark level, and pathological T stage according to the 8th edition of the AJCC TNM classification. Tissue samples were obtained from primary melanomas at surgical resection, before the subsequent development of distant metastases. Lesions were diagnosed based on histopathological examination of formalin-fixed paraffin-embedded tissue sections. Patients were retrospectively classified according to metastatic status during follow-up.

Peripheral blood samples were obtained from patients at the Department of Dermatology, Faculty of Medicine, University of Debrecen, Hungary. Metastatic status was based on PET-CT, CT, or soft-tissue ultrasound performed within three months before blood collection. The lung-metastasis group included patients for whom the lungs were the only distant metastatic site at blood sampling, whereas the other-distant-metastasis group included patients with metastases at sites other than the lungs and no detectable lung metastases. The study was approved by the Ethics Committee of the Hungarian Scientific Council for Health [document numbers: TUKEB 17876–2018/EKU (date of approval: 28 August 2018) and BMEU/715-1/2022/EKU (date of approval: 4 July 2022)] and was performed according to the relevant guidelines. The clinicopathological parameters of the melanoma tissues and plasma samples are summarized in Table 2.

### 2.3. In Vitro Invasion Assay

We assessed the invasion of melanoma cells using the BD Biocoat Matrigel invasion assay (pore size: 8 μm, 24 wells; BD Biosciences, Bedford, MA, USA) as previously described [18]. Briefly, melanoma cells were seeded into the upper chambers in serum-free culture medium. Complete endothelial culture medium containing 10% fetal bovine serum (FBS) was used in the lower chambers as the control condition, whereas the experimental condition consisted of 50% HPMEC-conditioned medium and 50% fresh, complete endothelial culture mediumAfter 24 h of incubation at 37 °C, the invasive cells that had penetrated and adhered to the bottom layer of the Matrigel membrane were fixed, stained, and then counted at 200× magnification in seven different fields of view using a Zeiss Primovert inverted microscope (Carl Zeiss AG, Oberkochen, Germany).

The invasion assays were performed in three independent biological experiments, with three independent control and three independent HPMEC-CM-treated measurements obtained for each melanoma cell line. In each experiment, invading cells were counted in seven microscopic fields per insert, and the field counts were averaged to obtain a single invasion measurement per replicate. Invasion results were expressed as percentages and compared between control and HPMEC-CM-treated conditions for each melanoma cell line.

### 2.4. Co-Culturing of Melanoma Cell Lines and Endothelial Cells

For the co-culture, we used BD Biocoat Matrigel invasion chambers (pore size: 8 μm, 6 wells; BD Biosciences, Bedford, MA, USA) as we previously described [18]. Briefly, melanoma cells (2 × 10^5^ cells/well) were added to the upper chamber, which was then carefully inserted into six-well plates coated with a monolayer of HPMECs. The cells were co-incubated for 24 h, after which the insert containing the melanoma cells was transferred to a new plate. The invading melanoma cells and the non-invading cells were treated separately with a 0.5% trypsin/0.2% EDTA solution (Sigma-Aldrich Inc., St. Louis, MO, USA) to detach them from the membrane and then cultured according to standard protocols.

### 2.5. Real-Time Quantitative PCR

RNA extraction was performed using the NucleoSpin RNA kit (Macherey–Nagel, Dueren, Germany) according to the manufacturer’s instructions. RNA concentration and quality were measured using a NanoDrop instrument (Agilent Technologies, Palo Alto, CA, USA). RNA samples with a 260/280 ratio ≥1.8 were retained for further analysis. Reverse transcription of total RNA (1000 ng) was performed using the High-Capacity cDNA Archive Kit (Applied Biosystems, Carlsbad, CA, USA) according to the manufacturer’s instructions.

We determined the relative expression levels of 96 genes (20 chemokine receptor genes, 43 interleukin receptor genes, 25 tumor necrosis factor receptor genes, and 8 housekeeping genes) using a LightCycler^®^ 480 Real-Time PCR System (Roche Diagnostics, GmbH, Mannheim, Germany). The primer sets (Human Cytokine and Chemokine Receptor Primer Library, RealTimePrimers.com, Elkins Park, PA, USA) were used as previously described [19]. The data are presented as the mean of 2^−ΔCt^ values from three independent experiments.

### 2.6. Proteome Profiler Assay

RIPA Lysis and Extraction Buffer (Thermo Fisher Scientific Inc., Waltham, MA, USA) containing 20 µL protease and phosphatase inhibitor cocktail (Thermo Fisher Scientific Inc., Waltham, MA, USA) was used for protein extraction, and the concentration was determined using the Pierce™ Coomassie (Bradford) Protein Assay Kit (Thermo Fisher Scientific Inc., Waltham, MA, USA). The Proteome Profiler Human Chemokine Array Kit was used to determine the expression of 31 different chemokines (R&D Systems, Inc., Minneapolis, MN, USA). We used all necessary reagents and the microarray procedure in accordance with the manufacturer’s detailed protocol. The target proteins were detected and visualized using the Azure c300 chemiluminescent imaging system (Dublin, CA, USA) with the Chemi Reagent Mix (R&D Systems Inc., Minneapolis, MN, USA). Data analysis was performed using AzureSpot software (version 2.2.167). The intensity of the positive control (reference spot) was set to 100%.

### 2.7. Enzyme-Linked Immunosorbent Assay (ELISA)

Plasma concentrations of IL-8 (cat. no. HUFI00184), GROα (cat. no. HUFI00005), Midkine (cat. no. HUFI00202), and IL-11 (cat. no. HUFI00157) were measured using commercially available human ELISA kits from Assay Genie (Dublin, Ireland). Soluble RANKL (sRANKL; cat. no. ELK10470) and CCL2 (cat. no. ELK5252) were measured using ELISA kits from ELK Biotechnology. Plasma samples were diluted 1:2 for the Assay Genie kits and 1:4 for the ELK Biotechnology kits. All assays were performed according to the manufacturers’ instructions. Samples were analyzed in duplicate, and absorbance was measured at 450 nm using an Epoch™ Microplate Spectrophotometer (BioTek Instruments, Winooski, VT, USA). Standard curves were generated for each plate, and sample concentrations were calculated based on the corresponding standard curves.

### 2.8. Statistical Analysis

Statistical analyses were performed using IBM SPSS Statistics version 31 (IBM Corp., Armonk, NY, USA). Data normality was assessed using the Shapiro–Wilk test. Differences between two independent groups were evaluated using the Mann–Whitney U test, whereas comparisons involving three groups were performed using the Kruskal–Wallis test, followed, where appropriate, by pairwise comparisons with Bonferroni-adjusted *p*-values. A *p*-value ≤ 0.05 was considered statistically significant.

For the invasion assays, three independent biological experiments were performed per melanoma cell line and experimental condition, with the mean number of invading cells across seven microscopic fields representing one biological replicate. Given the small sample size, both asymptotic and exact two-sided *p*-values were reported, with the exact results used for statistical interpretation.

For the receptor-expression analyses, individual biological replicates were initially compared in an exploratory analysis. To account for repeated measurements within melanoma cell lines, expression ratios were additionally averaged within each cell line, and the resulting values were compared between the increased- and unchanged-invasiveness groups (*n* = 3 independent cell lines per group). Exact two-sided *p*-values from these cell-line-level comparisons were used for statistical inference.

## 3. Results

### 3.1. Effect of HPMEC-CM on Melanoma Cell Invasion

**Figure 1:**
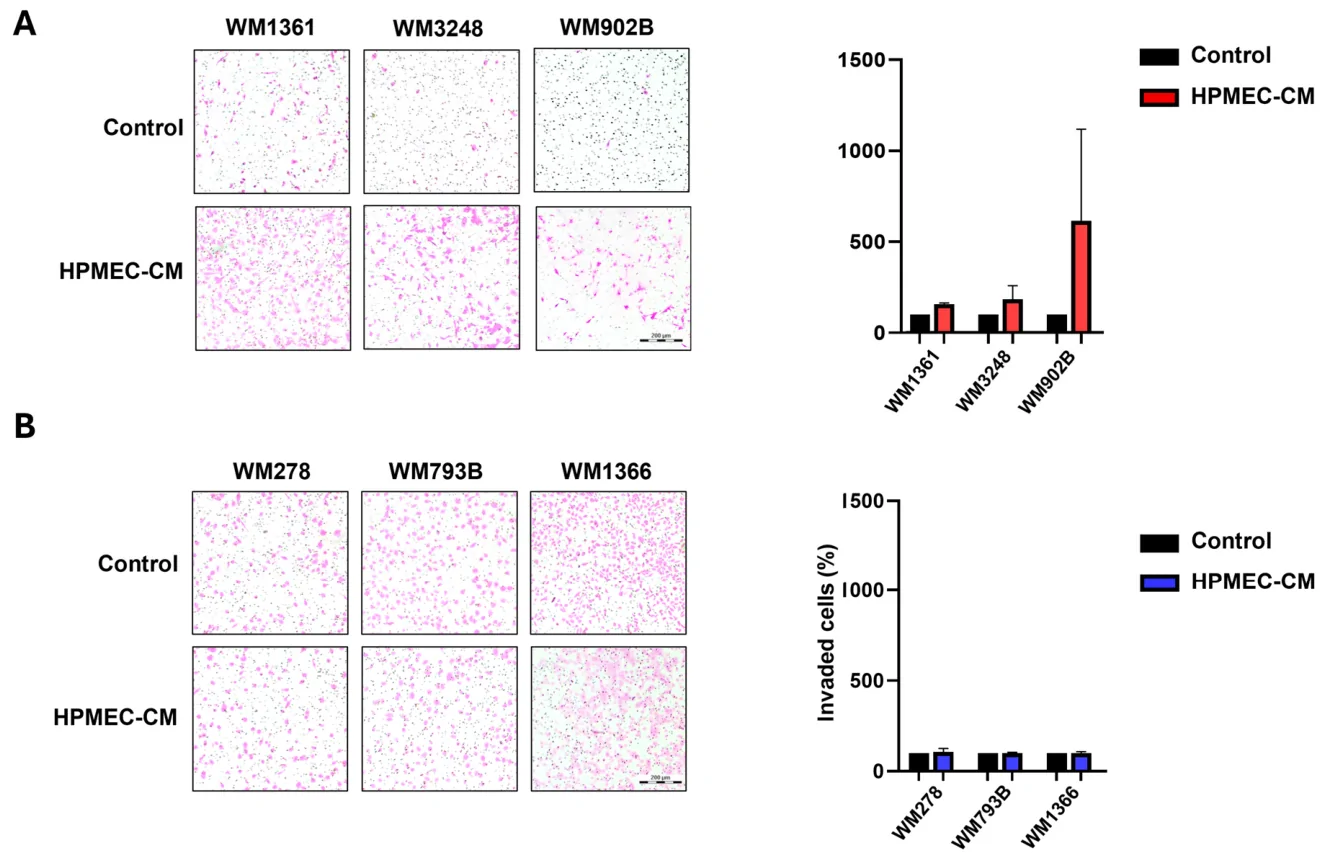
Invasive responses of melanoma cell lines to HPMEC-conditioned medium (HPMEC-CM). Complete, unconditioned endothelial culture medium supplemented with 10% FBS was used as the control, whereas the experimental condition consisted of 50% HPMEC-CM and 50% fresh endothelial culture medium. (**A**) Representative images of the invasive cells at 50× magnification showing increased invasion in the WM1361, WM3248, and WM902B cell lines. (**B**) Representative images of the invasive cells at 50× magnification, showing no comparable increase in invasion in the WM278, WM793B, and WM1366 melanoma cell lines. Three independent biological measurements were obtained for each condition and cell line. Invading cells were counted in seven microscopic fields per insert, and the results were expressed as percentages. Differences between control and HPMEC-CM-treated conditions were evaluated separately for each melanoma cell line using the Mann–Whitney–Wilcoxon test (asymptotic *p*-values = 0.037); however, these differences did not reach statistical significance when exact two-sided tests were applied (*p* = 0.100).

To investigate receptor-expression patterns associated with the differential invasive responses of melanoma cells to the pulmonary endothelial environment, the six melanoma cell lines were first classified according to their responses to HPMEC-CM using unconditioned endothelial culture medium with 10% fetal bovine serum (FBS) as a chemoattractant (control condition). Three melanoma cell lines (WM1361, WM3248, and WM902B) exhibited increased invasion in response to HPMEC-CM compared with control conditions, although these differences did not reach statistical significance according to the exact two-sided Mann–Whitney U test (Figure 1).

### 3.2. Chemokine and Cytokine Receptor Expression of Melanoma Cell Lines

To investigate the gene-expression differences induced by HPMEC, the melanoma cell lines were classified into two groups based on their invasive response: a non-responsive group, which showed no change (WM278, WM793B, and WM1366), and a responsive group (WM902B, WM1361, and WM3248), which exhibited increased invasiveness. Subsequently, a separate HPMEC co-culture invasion model was used to isolate invasive and non-invasive melanoma cell subpopulations from each cell line using Matrigel invasion chambers. In this way, we established six invasive and six non-invasive subpopulations. These populations were then subjected to chemokine and cytokine receptor-expression analysis to identify molecular differences associated with their invasive phenotypes.

**Figure 2:**
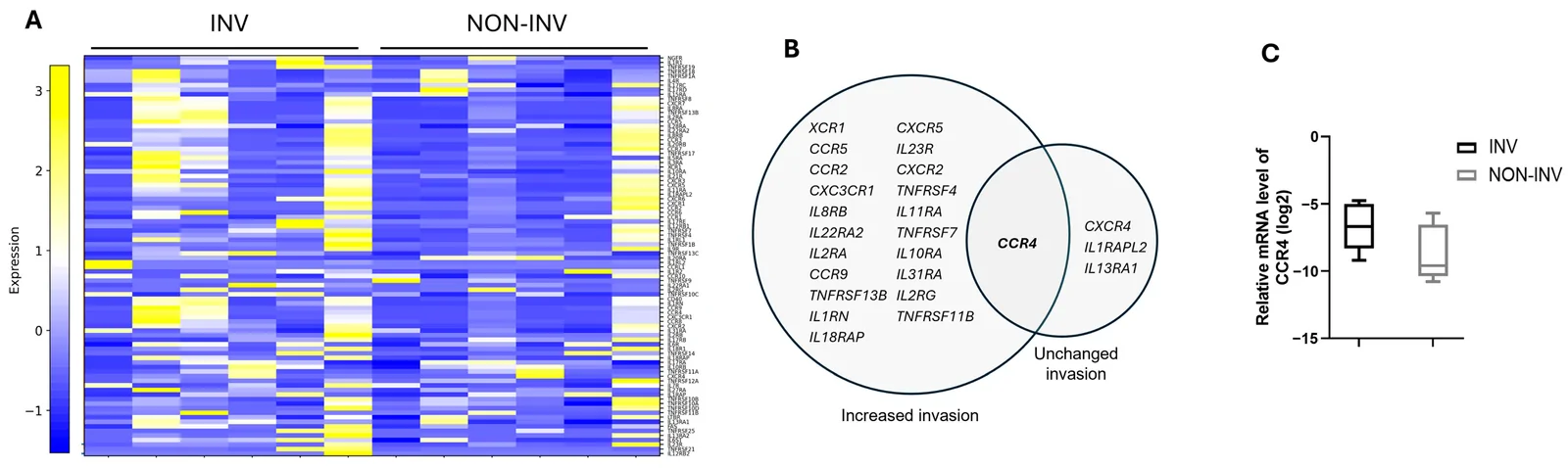
Relative expression levels of chemokine and cytokine receptors in melanoma cells. (**A**) Heatmap showing expression profiles of 88 chemokine and cytokine receptor genes in the selected invasive (INV) and non-invasive (NON-INV) melanoma cell populations. Cell populations are shown vertically, whereas genes are displayed horizontally. The heatmap was generated using https://heatmapper2.ca/ [20] accessed on 7 May 2026. (**B**) Venn diagram showing the genes that were upregulated in the invasive cell population compared with the corresponding non-invasive populations in melanoma cell lines with increased invasiveness (left side; WM902B, WM1361, and WM3248) and those with unchanged invasive capacity (right side; WM278, WM793B, and WM1366). *CCR4* was located in the overlapping region, indicating elevated expression in the invasive subpopulations of both groups. (**C**) Relative CCR4 mRNA expression level (log_2_-transformed data) in the invasive and non-invasive cell populations.

Gene expression profiling of chemokine and cytokine receptors revealed distinct patterns between the six selected invasive and non-invasive subpopulations (Figure 2A). For each cell line, we calculated the ratio of relative expression between the invasive and non-invasive cell populations as described previously [18].

Based on these ratios, 22 genes were consistently upregulated in the invasive subpopulations of all cell lines that exhibited increased invasiveness (WM902B, WM1361, and WM3248) compared with their corresponding non-invasive cells (Figure 2B; left side of the Venn diagram). In contrast, among the cell lines with unchanged invasiveness (WM278, WM793B, and WM1366), only four genes were consistently upregulated in the invasive subpopulation relative to the non-invasive cells (Figure 2B; right side of the Venn diagram). Notably, CCR4 expression was consistently higher in the invasive subpopulations regardless of the HPMEC-CM-associated invasive phenotype; however, the relative difference in expression between invasive and non-invasive cells did not reach statistical significance (Figure 2C).

Comparison of INV/NON-INV expression ratios revealed higher mean values for seven candidate genes in melanoma cell lines exhibiting increased invasiveness compared with those showing unchanged invasiveness (Table 3). Although exploratory replicate-level comparisons suggested differences between the groups, none reached statistical significance when the independent melanoma cell lines were used as the units of analysis (exact two-sided Mann–Whitney U test, *p* = 0.100–0.200).

To further investigate the relationship between receptor expression and invasive behavior, correlation analyses were performed between the percentage of invasive cells (%) and relative gene expression levels. Significant positive correlations were identified for CCR2, IL11RA, IL15RA, TNFRSF11B, and TNFRSF12A, with correlation coefficients ranging from 0.829 to 0.999 (*p* < 0.05), indicating that higher expression of these receptors was associated with greater melanoma cell invasiveness.

### 3.3. Chemokine and Cytokine Receptor Expression of Melanoma Tumor Samples

To validate our in vitro findings, we analyzed a small cohort of primary melanoma tissue samples comprising non-metastatic tumors (*n* = 3), primary melanomas from patients who subsequently developed lung metastasis (*n* = 6), and primary melanomas from patients who developed metastases to other distant organs (*n* = 6). First, we evaluated *CCR4* gene expression, as our in vitro experiments consistently demonstrated its upregulation in invasive melanoma cells. Although *CCR4* expression was higher in primary tumors from patients with metastatic disease than in those without metastasis, it did not reach statistical significance (*p* = 0.204; Supplementary Figure S1).

**Figure 3:**
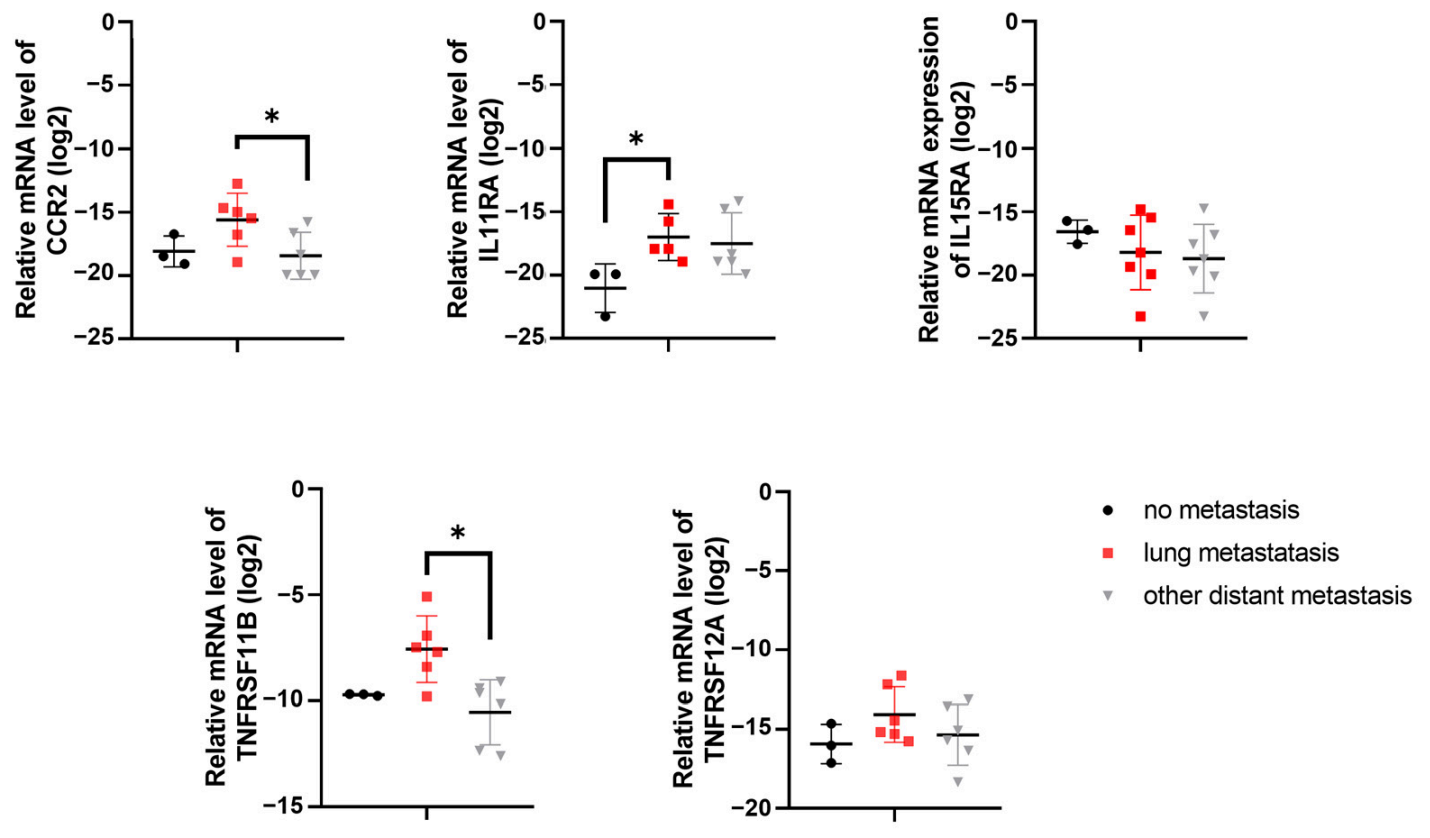
Relative expression of candidate chemokine and cytokine receptors in primary melanoma tumor samples. Gene expression levels were compared among primary melanomas without metastasis (black; *n* = 3), primary melanomas from patients with lung metastases (red; *n* = 6), and primary melanomas from patients with metastases to other distant organs (grey; *n* = 6). Data are presented as the mean ± standard deviation (SD). Statistical differences among the three groups were assessed using the Kruskal–Wallis test; where the overall test was significant, pairwise comparisons were performed with Bonferroni adjustment. Asterisks indicate significant pairwise differences based on Bonferroni-adjusted *p*-values (* *p* ≤ 0.05).

In addition, we evaluated the gene expression of candidate genes (*CCR2*, *IL11RA*, *IL15RA*, *TNFRSF11B*, and *TNFRSF12A*; Figure 3) in primary melanoma tumor samples. *CCR2* and *TNFRSF11B* expression levels were significantly higher in primary tumors from patients with lung metastases than in those from patients with metastases to other distant organs. Furthermore, *IL11RA* expression was significantly higher in primary tumors from patients with lung metastasis than in non-metastatic primary tumors.

### 3.4. Chemokine and Cytokine Expression of HPMECs

**Figure 4:**
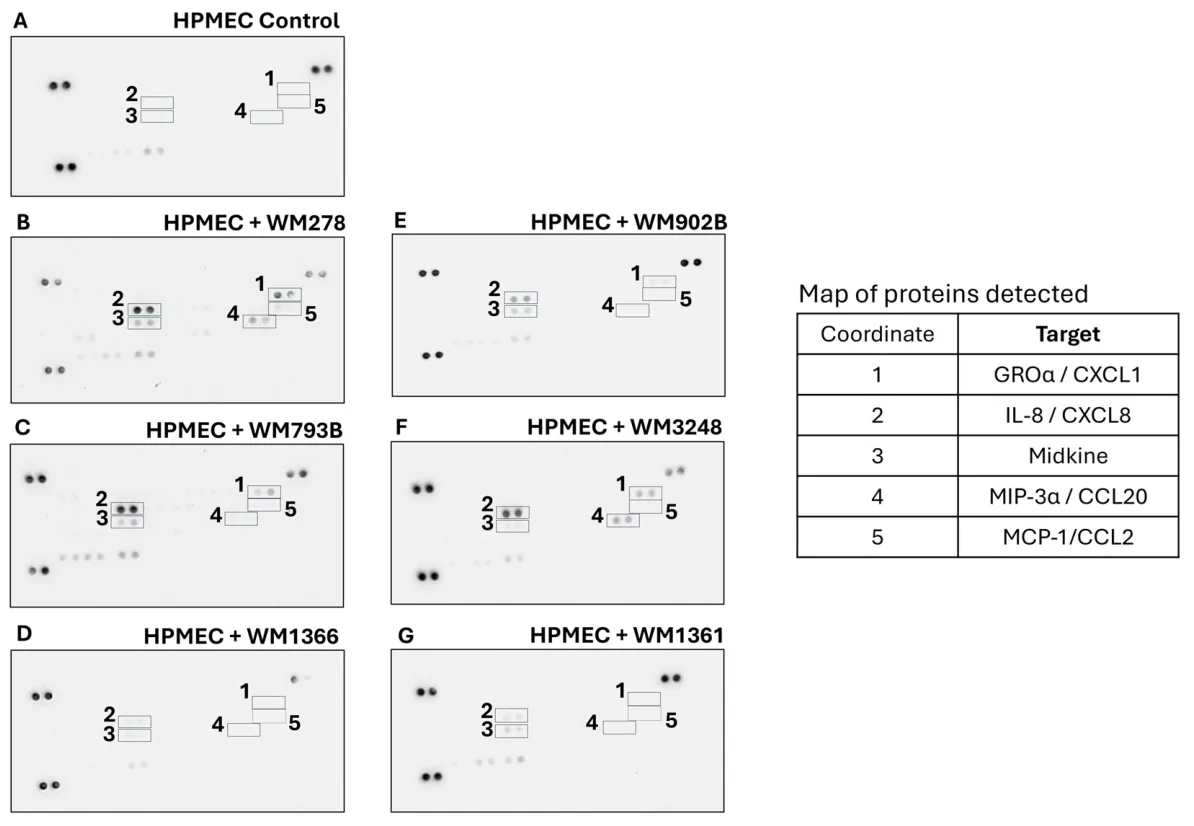
Chemokine protein expression profile of human pulmonary microvascular endothelial cells (HPMEC) determined using the Proteome Profiler Human Chemokine Array. Representative arrays of HPMECs cultured (**A**) alone (control) or following co-culture with the melanoma cell lines, including (**B**) WM278, (**C**) WM793B, (**D**) WM1366, (**E**) WM902B, (**F**) WM3248, or (**G**) WM1361. The array map identifies the position of each of the 31 chemokines. Each chemokine is represented by duplicate spots on the membrane.

To characterize the baseline chemokine expression profile of HPMECs and changes following exposure to melanoma cells, we analyzed endothelial-cell lysates using the Proteome Profiler Human Chemokine Array, which detects 31 chemokines. Untreated HPMECs were used as controls and compared with HPMECs co-cultured with melanoma cell lines. Endothelial cells were subsequently classified into two groups according to whether they had been co-cultured with melanoma cell lines that exhibited either increased invasiveness or unchanged invasiveness. No distinct chemokine expression pattern was observed between these two groups. However, comparison with the control endothelial cell profile revealed that IL-8 expression was increased after co-culturing with all six melanoma cell lines (Figure 4). In addition, GROα expression was also elevated in HPMECs after co-culture with four of the six melanoma cell lines. Midkine expression was induced in endothelial cells in response to three of the six melanoma cell lines. MIP-3α and MCP-1 were detected in two and one co-culture conditions, respectively. None of these chemokines were detected in untreated control endothelial cells.

### 3.5. Chemokine and Cytokine Concentration in Plasma Samples of Melanoma Patients

Based on the endothelial chemokine expression profile, plasma concentrations of IL-8, GROα, and Midkine were determined in melanoma patients who had no metastases, with lung metastases, and with metastases to other distant organs. In addition, CCR2 and IL11RA were upregulated in invasive melanoma cells; their respective ligands, CCL2 and IL-11, were also included in the plasma analysis. Soluble RANKL was also measured due to its functional interaction with TNFRSF11B/osteoprotegerin within the RANK–RANKL–OPG axis. The plasma concentrations of all six proteins overlapped substantially among the three patient groups, and no statistically significant differences were detected using the Kruskal–Wallis test (Supplementary Figure S2). Although elevated concentrations were observed in individual patients for several proteins, these increases were inconsistent and did not distinguish patients with lung metastases from the other clinical groups. Some CCL2 measurement results exceeded the upper limit of quantification. For statistical analysis, we assigned the upper limit of quantification for this study to these results; therefore, the values shown in the graph should not be interpreted as the exact concentrations of these samples but rather as concentrations at or above the upper limit of quantification.

## 4. Discussion

Metastasis is increasingly recognized as a dynamic process driven by reciprocal interactions between tumor cells and their microenvironment, rather than solely by the intrinsic properties of tumor cells [21,22]. Although endothelial cells have traditionally been regarded as passive barriers to metastatic dissemination, accumulating evidence indicates that they actively promote tumor cell extravasation, invasion, and colonization through the secretion of cytokines, chemokines, and growth factors [10,23]. Consistent with this concept, our findings demonstrate that conditioned medium derived from human HPMECs enhanced the invasive capacity of several melanoma cell lines, highlighting the marked biological heterogeneity in melanoma. Previous studies have demonstrated that melanoma cells exhibit substantial genetic heterogeneity and phenotypic plasticity, resulting in differential responsiveness to stromal-derived cytokines, growth factors, and other microenvironmental signals [24,25]. Consistent with this heterogeneity, we observed different invasive responses to HPMEC-CM among the melanoma cell lines. Based on these responses, the cell lines were classified into two groups, providing a basis for comparing receptor-expression patterns in invasive and non-invasive subpopulations subsequently isolated using a separate HPMEC co-culture model. Although the experimental design does not distinguish the effects of individual endothelial-derived factors from other conditioning-related changes in medium composition, it allowed us to characterize differences in melanoma-cell responses to HPMEC-CM. Furthermore, potential contributions of cell proliferation or survival to the number of cells traversing the Matrigel-coated membrane during the 24-h assay cannot be completely excluded. Importantly, because the HPMEC-CM and direct co-culture experiments represent distinct experimental conditions, whether the identified receptor-expression patterns are also observed following exposure to HPMEC-CM remains to be determined.

Comparison of invasive and non-invasive subpopulations revealed distinct expression patterns between melanoma cell lines that exhibited increased invasion in response to HPMEC-CM and those that did not. Whereas only four genes were consistently upregulated in the invasive subpopulations of melanoma cell lines with unchanged invasive capacity, 22 genes were upregulated in the invasive subpopulations of melanoma cell lines that responded to HPMEC-CM with enhanced invasion. Among these, *CCR4* was the only gene consistently expressed at higher level in invasive cells, regardless of their response to HPMEC-CM. Although this increase in *CCR4* expression did not reach statistical significance, its consistent upregulation in invasive populations suggests that *CCR4* may be associated with the invasive phenotype rather than specifically with responsiveness to pulmonary endothelial-derived signals. Previous studies have implicated *CCR4* in melanoma brain metastasis [26,27], while more recent evidence has highlighted the role of the CCL17/CCR4 signaling axis in tumor cell migration, invasion, immunomodulation, and metastatic progression across a range of malignancies [28,29,30,31].

In contrast, *CCR2*, *IL11RA*, *IL15RA*, *TNFRSF11B*, and *TNFRSF12A* showed expression patterns that may be associated with the invasive phenotype in the HPMEC-based experimental models. Although exploratory analyses suggested differences between the invasion-response groups, these differences were not statistically significant when independent melanoma cell lines were used as the units of analysis. Nevertheless, the observed expression patterns identify these receptors as candidates for further investigation. Their potential functional involvement in melanoma invasion remains to be established. Among these candidates, the CCR2/CCL2 signaling axis has been extensively implicated in tumor progression through multiple mechanisms, including the promotion of cell proliferation, migration, and invasion; induction of epithelial–mesenchymal transition (EMT); stimulation of vascular endothelial growth factor (VEGF) production and tumor angiogenesis, recruitment of tumor-associated cells to the tumor microenvironment, and remodeling of the tumor stroma to create an immunosuppressive microenvironment [11,19,32,33].

Likewise, IL-11/IL11RA signaling has been shown to facilitate metastatic colonization through activation of the JAK/STAT3 pathway, thereby promoting tumor cell survival and outgrowth at distant metastatic sites [34]. Although the role of IL-11/IL11RA signaling has been investigated predominantly in the context of bone and liver metastasis, our findings identify IL11RA as a candidate receptor associated with the expression patterns observed in our experimental and tissue analyses [18,35,36,37]. Similarly, TNFRSF11B (osteoprotegerin; OPG) has been extensively studied in bone biology primarily for its role as a decoy receptor that regulates RANKL signaling. However, accumulating evidence indicates that OPG also contributes to tumor progression through mechanisms involving endothelial activation and resistance to apoptosis [38,39,40]. Our findings raise the possibility that OPG-related signaling may warrant further investigation in the context of pulmonary melanoma metastasis.

Likewise, TNFRSF12A (Fn14), the receptor for TWEAK, has been implicated in tissue remodeling, inflammatory signaling, and tumor invasion. Activation of the TWEAK/Fn14 signaling pathway has been associated with increased tumor aggressiveness, epithelial–mesenchymal transition, and metastatic progression in several malignancies [41,42,43]. These observations identify *TNFRSF12A* as another candidate receptor of interest in melanoma–endothelial interactions Validation in primary melanoma tissue samples provided preliminary clinical support for our in vitro findings. Despite the limited cohort size, *CCR2* and *TNFRSF11B* expression levels were significantly higher in primary tumors from patients who subsequently developed lung metastases than those from patients who developed metastases to other distant organs, whereas *IL11RA* expression was significantly elevated compared with that in non-metastatic primary tumors. Although these observations require validation in larger, independent patient cohorts, it suggest that receptor-expression patterns identified in the experimental model may also be associated with differences in metastatic among patients with primary melanomas Gene-expression analysis of primary melanoma tissues represents a composite molecular profile of the tumour rather than melanoma-cell-specific expression. Bulk tumour tissue contains malignant, immune, stromal, and vascular components, and variation in these compartments may contribute to the observed expression differences. Accordingly, the identified associations should be interpreted as tumour-tissue-level molecular patterns associated with subsequent metastatic outcome, without assigning individual receptor expression to a specific cellular compartment. Importantly, the candidate signaling systems examined here are not restricted to tumor biology but also have established roles in immune regulation and tissue homeostasis. The CCL2/CCR2 axis contributes to inflammatory monocyte recruitment in the lung and may also influence pulmonary endothelial barrier function [44,45]. IL-11/IL11RA signaling participates in pulmonary stromal responses and tissue remodeling, including regulation of fibroblast activation and extracellular matrix organization [46]. The RANK/RANKL/OPG system is best characterized in bone remodeling and immune regulation, although emerging evidence suggests that OPG may also influence the pulmonary metastatic microenvironment through immune-cell-dependent mechanisms [47].

To extend the findings regarding receptor expression to the corresponding soluble ligands, we measured the plasma concentrations of CCL2, IL-11, and soluble RANKL in patients with melanoma. These analyses were exploratory in nature and were not intended to confirm the activation of the corresponding receptor–ligand systems in the HPMEC invasion model Although none of these ligands differed significantly among the clinical groups, these findings do not necessarily diminish the biological relevance of the corresponding receptor–ligand axes. Endothelial activation is widely recognized as an early event in pre-metastatic niche formation, promoting cytokine secretion, increased vascular permeability, enhanced leukocyte recruitment, extracellular matrix remodeling, and the expression of endothelial adhesion molecules that facilitate tumor cell extravasation [10,11,12]. In this context, receptor expression may provide more biologically relevant information than circulating ligand concentrations, as it reflects the capacity of melanoma cells to recognize and respond to organ-specific microenvironmental signals. Moreover, circulating plasma concentrations may not accurately reflect local or transient cytokine concentrations within the pulmonary endothelial microenvironment, where receptor–ligand interactions are likely to occur.

Our findings also indicate that co-culture with melanoma cells is associated with changes in the chemokine expression profile of pulmonary endothelial cells. IL-8 expression was consistently increased in endothelial cells following co-culture with all melanoma cell lines examined, indicating that endothelial activation may occur independently of the invasive phenotype of melanoma cells. Interleukin 8 (IL-8; also known as CXCL8) is one of the best-characterized pro-inflammatory chemokines involved in angiogenesis, endothelial activation, neutrophil recruitment, and tumor progression [48,49]. Under physiological conditions, IL-8 is expressed at very low levels, but can be rapidly induced in response to inflammatory and tumor-derived stimuli [48,50]. Increasing evidence has linked IL-8 to epithelial–mesenchymal transition (EMT), promoting tumor cell plasticity, migration, and transendothelial invasion, and thereby facilitating metastatic dissemination [48,51,52]. Furthermore, IL-8 has been implicated in the establishment of the pre-metastatic niche by promoting inflammatory cell recruitment and increasing vascular permeability, thereby creating a microenvironment that supports tumor cell extravasation and subsequent metastatic colonization [7].

Some limitations of the present study should be acknowledged. The number of melanoma cell lines and patient samples was relatively small, particularly in the tissue validation cohort. Although conditioned medium experiments enabled us to investigate soluble endothelial-derived signals, they did not account for direct cell–cell interactions or extracellular matrix components that also contribute to metastatic colonization in vivo. Future studies incorporating receptor-specific perturbation with organ-specific endothelial co-culture models will be necessary to establish the mechanistic contribution of the identified signaling pathways. “Moreover, the present in vitro models address melanoma invasion in response to HPMEC -associated conditions but do not reproduce vascular extravasation, survival in the circulation, pulmonary colonization, metastatic outgrowth, or pre-metastatic niche formation in vivo. Accordingly, the relevance of the identified associations to these later stages of metastasis will require investigation in appropriate experimental models.

## 5. Conclusions

In summary, conditioned medium derived from HPMECs influenced melanoma cell invasion in different ways, and HPMEC-based experimental models identified characteristic receptor expression patterns associated with the invasive phenotype. CCR2, IL11RA, TNFRSF11B, and TNFRSF12A emerged as candidate receptors of interest, while tissue analyses further supported association between the expression of CCR2, IL11RA, and TNFRSF11B and metastatic outcomes involving the lung. Taken together, these results reveal molecular associations between melanoma invasion and the pulmonary endothelial microenvironment and provide a basis for future studies investigating the functional roles of these receptor–ligand systems.

## Figures and Tables

**Table 1:** Clinicopathological characteristics of human primary melanoma cell lines.

Cell Line	Growth Phase ^1^	Histological Subtype ^2^	*BRAF* Mutation Status ^3^	*NRAS* Mutation Status ^4^
WM793B	RGP/VGP	SSM	V600E	WT
WM1361	VGP	SSM	WT	Q61L
WM278	VGP	NM	V600E	WT
WM902B	VGP	n.d.	V600E	WT
WM1366	VGP	SSM	WT	Q61L
WM3248	VGP	n.d.	V600E	WT

^1^ RGP: radial growth phase; VGP: vertical growth phase; ^2^ SSM: superficial spreading melanoma; NM: nodular melanoma; n.d.: data not available; ^3^ V: valine; E: glutamic acid; WT: wild type; ^4^ Q: glutamine; and L: leucine.

**Table 2:** Clinicopathological parameters of melanoma tissue and plasma samples.

Variables	N	Variables	N
Primary melanoma tumor samples	15	Primary melanoma plasma samples	10
Gender		Gender	
Male	10	Male	6
Female	5	Female	4
Age (years)		Age (years)	
20–50	2	20–50	2
>50	13	>50	8
Breslow thickness		Breslow thickness	
<4.00	6	<4.00	8
≥4.00	8	≥4.00	2
No data	1		
Subtype ^1^		Subtype ^1^	
SSM	10	SSM	6
NM	5	NM	4
Metastasis formation ^2^		Metastasis formation ^2^	
Non-metastatic	3	Non-metastatic	10
Lung metastasis	6	Distant organ metastasis	0
Other distant organ	6		
Ulceration		Ulceration	
Present	11	Present	4
Absent	4	Absent	6
		Metastatic melanoma plasma samples	20
		Gender	
		Male	7
		Female	13
		Age (years)	
		20–50	4
		>50	16
		Localization	
		Lung	10
		Other distant organ	10

^1^ SSM: superficial spreading melanoma, NM: nodular melanoma, LM: lentigo melanoma; ^2^ Patients with follow-up periods of 36 months were included in the study.

**Table 3:** Relative gene expression ratios (invasive vs. non-invasive) of chemokine and cytokine receptors in melanoma cells.

Gene Symbol	Fold Change (Mean ± SD) ^1^		Replicate Level *p* ^4^	Cell-Line-Level *p* ^5^
	Increased Invasiveness ^2^	Unchanged Invasiveness ^3^		
*CCR2*	199.3 ± 364.1	1.2 ± 1.4	**0.021**	**0.100**
*IL2RG*	6.1 ± 7.1	1.4 ± 1.7	**0.009**	**0.100**
*IL11RA*	21.6 ± 29.8	2.8 ± 2.3	**0.017**	**0.100**
*IL15RA*	44.5 ± 93.6	1.1 ± 0.8	**0.049**	**0.100**
*IL22RA2*	41.9 ± 63.9	2.4 ± 3.9	**0.014**	**0.100**
*TNFRSF11B*	10.8 ± 22.6	1.2 ± 0.5	**0.021**	**0.100**
*TNFRSF12A*	3.2 ± 4.9	0.6 ± 0.4	**0.009**	0.200

^1^ Gene expression results are presented as the mean ratio of the untransformed 2^−ΔCt^ values between invasive and non-invasive populations from three independent biological experiments and summarized for each melanoma cell line; ^2^ cell lines exhibiting increased invasiveness in response to HPMEC-CM (WM902B, WM1361, and WM3248); ^3^ cell lines showing no change in invasiveness (WM278, WM793B, and WM1366); ^4^ Exploratory *p*-values were calculated using exact two-sided Mann–Whitney U tests on individual biological replicates, without accounting for within-cell-line dependence; ^5^ Cell-line-level *p*-values were calculated using exact two-sided Mann–Whitney U tests on mean expression ratios from the melanoma cell lines per group.

## Data Availability

The original contributions presented in this study are included in the article/Supplementary Material. Further inquiries can be directed to the corresponding author(s).

## Supplementary Materials

The following supporting information can be downloaded at https://www.mdpi.com/article/10.3390/biom16101510/s1, Figure S1: Relative expression of the *CCR4* gene in primary melanoma tumor samples. The qRT-PCR results are presented as the mean of 2^−ΔCt^ values. ns: no significant difference between primary tumors without metastasis and those with distant organ metastasis (Mann–Whitney–Wilcoxon test).; Figure S2: Chemokine and Cytokine Concentration in Plasma Samples of Melanoma Patients. Plasma levels were compared among patients without metastasis (black; *n* = 10), with lung metastases (red; *n* = 10), and with metastases to other distant organs (grey; *n* = 10). Open symbols in the CCL2 panel indicate samples with concentrations exceeding the upper limit of detection. Data are presented as means of duplicates. No statistically significant differences were observed among the groups (Kruskal–Wallis test).

## Abbreviations

The following abbreviations are used in this manuscript:

PMN	Pre-metastatic niche
TGF-ß	Transforming growth factor-β
TNF	Tumor necrosis factor
CCL2	Chemokine C-C motif ligand 2
BMDCs	Bone marrow-derived cells
CTCs	Circulating tumor cells
HPMECs	Human pulmonary microvascular endothelial cells
CM	Conditioned media
EMT	Epithelial–mesenchymal transition
VEGF	Vascular endothelial growth factor
OPG	Osteoprotegerin
